# Neonicotinoids: insecticides or a threat to public health?

**DOI:** 10.3389/fpubh.2026.1816900

**Published:** 2026-04-09

**Authors:** Cesar Soria-Fregozo, Silvia Lizette Ramos de Robles, Mario Eduardo Flores-Soto, Gilberto Uriel Rosas-Sánchez

**Affiliations:** 1 Departamento de Ciencias de la Tierra y de la Vida, Centro Universitario de Los Lagos, Universidad de Guadalajara, Lagos de Moreno, Mexico; 2 Centro Universitario de Ciencias Biológicas y Agropecuarias, Zapopan, Mexico; 3 Laboratorio de Neurobiología Celular y Molecular, División de Neurociencias, Centro de Investigación Biomédica de Occidente (CIBO), Instituto Mexicano del Seguro Social, Guadalajara, Mexico; 4 Programa de Estancias Posdoctorales por México, Centro Universitario de los Lagos, Universidad de Guadalajara, Lagos de Moreno, Mexico

**Keywords:** agricultural chemicals regulation, neonicotinoids, neurotoxic effects, pesticide toxicity, pollinator decline

## 1 Introduction

Neonicotinoid insecticides, developed in the 1990s, are widely used in agriculture due to their effectiveness against insect pests (1). Their mechanism disrupts the insect nervous system by mimicking nicotine (2). When first introduced to the market, they were promoted as safe to handle and for consumers; therefore, they were widely accepted and soon became the best-selling insecticides internationally, displacing older-generation insecticides such as organophosphates, methylcarbamates, and pyrethroids (3). Neonicotinoids, along with fipronil (another systemic insecticide), constitute one-third of the global insecticide market. These insecticides are systemic, allowing distribution throughout the plant (4), enabling effective pest management strategies (5). Additionally, they have high mobility in soil, promoting contamination of groundwater and surface water through runoff (6). Importantly, concentrations detected in aquatic and terrestrial environments have been reported within ranges associated with sublethal and lethal effects in sensitive non-target organisms, indicating that their environmental presence is not merely incidental but toxicologically relevant (7, 8). Along with their systemic properties, neonicotinoids are relatively persistent, offering potential for long-term phytosanitary activity. The half-lives of these compounds under aerobic soil conditions can vary widely. For clothianidin, laboratory-derived estimates range from 148 to 6,931 days, with the upper extreme reflecting controlled conditions of optimal temperature and moisture designed for regulatory worst-case assessment rather than typical agricultural soils (4). Field dissipation half-life values for clothianidin are considerably shorter, with representative estimates generally reported between 148 and 1,155 days, depending on soil type, climate, and agronomic context (9).

Currently, their use is questionable due to environmental impact and potential public health risks (10). Research shows that environmental concentrations of neonicotinoids –including those commonly found in agricultural soils, water bodies, and pollen –overlap with levels experimentally demonstrated to impair the behavior, reproduction, and survival of non-target organisms, especially pollinators (7, 8). These findings suggest that field-realistic exposure, not just laboratory-derived doses, is associated with pollinator population declines (11). Consequently, some countries have regulated or banned neonicotinoid use (12), alongside concerns about human health implications (13). Recent studies have evidenced risks of neonicotinoid toxic effects to human health, including neurotoxicity, hepatotoxicity, immunotoxicity, genotoxicity, and reproductive system impairments (14) as well as neurodevelopmental immunotoxicity and inflammation of the central nervous system (15). While considered less toxic to mammals than organophosphates (16), preclinical and epidemiological studies show adverse effects from acute and chronic exposure (17). This raises a critical question: are neonicotinoids indispensable for crop protection, or do their environmental presence and health risks require reconsidering their role in modern agriculture?

This opinion piece is motivated by three concurrent developments that require critical synthesis: the rapid accumulation of epidemiological and mechanistic evidence on human health risks published since 2022 (13, 17); the growing regulatory asymmetry between jurisdictions that have restricted key neonicotinoids and those that continue to expand their use (18, 19); and the absence of an integrative framework that addresses human toxicity, ecological disruption, and agricultural transition within a One Health perspective. Together, these gaps justify a timely and critical reappraisal of neonicotinoids' role in contemporary agriculture and public health policy. This article critically examines the available evidence on neonicotinoid risks to human health and ecosystems, identifies the limitations of current regulatory frameworks, and discusses pathways toward safer agricultural alternatives, with the aim of informing ongoing scientific and policy debates.

## 2 Emerging evidence of impact on human health

Widespread environmental presence means human exposure occurs through ingestion, inhalation, and dermal absorption (17, 20). Despite initial beliefs of low mammalian toxicity, emerging evidence suggests neurological and metabolic health risks (16). Neurological effects are particularly concerning. Acute poisonings cause nicotinic-like symptoms, with Japanese studies linking exposure to finger tremors, impaired memory, and headaches (20). Imidacloprid binds to mammalian nicotinic acetylcholine receptors, causing harmful neuronal responses (21). Of particular mechanistic significance is its main mammalian metabolite, desnitro-imidacloprid, which forms through hepatic nitro-group reduction after oral exposure (21). Unlike the parent compound, whose selectivity for insect-type nAChRs is determined by the electronegativity of its nitroguanidine pharmacophore, desnitro-imidacloprid lacks this structural constraint and binds with much greater potency to mammalian nAChR subtypes, including the α4β2 and α7 receptors that are central to cognitive function, attention, and memory consolidation in the human brain (22, 23). This metabolic activation represents a qualitative shift in toxicological profile: the compound to which mammalian tissues are effectively exposed is pharmacologically distinct from, and more dangerous to humans than, the compound subject to regulatory evaluation. This distinction has critical implications for pre-market risk assessment, which currently evaluates parent compounds in isolation and does not require systematic toxicological characterization of major human metabolites. As a result, regulatory acceptable daily intakes and safety thresholds established for imidacloprid may significantly underestimate the actual neurotoxic risk associated with dietary and environmental exposure in humans (20, 22).

Metabolic concerns also exist. Cross-sectional studies have reported associations between neonicotinoid exposure and increased fasting glucose and obesity, particularly in females, although the observational design of these studies precludes causal inference (24). Vulnerable populations face greater risks. Epidemiological studies have reported associations between maternal neonicotinoid exposure and adverse birth outcomes, including elevated congenital heart disease incidence, though causal relationships remain to be established through prospective designs (17) and potential neurodevelopmental disruption in children (17). In rodent gavage models, behavioral changes in offspring exposed to imidacloprid during fetal and early postnatal periods are similar to those observed after nicotine exposure, suggesting shared developmental neurotoxicity mechanisms through nAChR pathways (25). However, these findings should be interpreted with caution in the human context, as gavage administration differs from dietary exposure routes, rodent cytochrome P450 metabolism of imidacloprid occurs at rates not directly comparable to humans, and neurodevelopmental timelines between species are not equivalent. Direct human epidemiological evidence for these specific neurodevelopmental endpoints remains limited. These findings demand continued research, particularly longitudinal epidemiological studies on vulnerable populations, enhanced toxicological surveillance, and human biomonitoring to assess health outcomes and inform evidence-based policies (17).

### 2.1 Neurotoxic and developmental effects

Neonicotinoids, including imidacloprid, are increasingly recognized for neurotoxic effects extending beyond insects to mammals, with concerns focusing on neurodevelopmental disorders, learning, and memory impairments (14, 26).

Rodent studies demonstrate that low-dose chronic imidacloprid exposure causes significant neurological impairments, including memory deficits and altered immune responses (27). Mammalian susceptibility following early developmental exposure leads to altered behavioral and biochemical profiles in adulthood (25). Maternal imidacloprid exposure produces behavioral alterations resembling nicotine exposure effects, indicating common developmental neurotoxicity mechanisms (25). Epidemiological research has reported associations between neonicotinoid exposure and cognitive decline in adults, findings that require confirmation through longitudinal study designs before causal conclusions can be drawn (28). Children are considered particularly vulnerable populations, with some studies detecting neonicotinoid metabolites in the urine of most children (17). At the neurological level, experimental evidence indicates that imidacloprid can dysregulate neurogenesis and impair cognitive function in animal models, and has been associated with molecular alterations that overlap with pathological features observed in Alzheimer's disease, including disrupted cholinergic signaling and impaired synaptic plasticity (29). However, it must be emphasized that these findings come from experimental models using defined doses and exposure durations that may not reflect human environmental exposure, and that Alzheimer's disease is a complex, multifactorial condition involving genetic, vascular, metabolic, and environmental determinants. The available evidence does not establish neonicotinoid exposure as a causative or sufficient factor in its development. These observations are best interpreted as preliminary mechanistic evidence warranting further investigation, not as a demonstrated link between neonicotinoid exposure and clinical Alzheimer's disease. Its presence has been detected in cerebrospinal fluid (30), along with a decrease in brain antioxidant enzymes (31), alteration of cell signaling, impairment of sensorimotor performance, and disruption of brain development in early stages (32).

#### 2.1.1 Mechanisms of neurotoxicity

Neurotoxic effects are attributed to several interconnected mechanisms. Neonicotinoids act as agonists at nicotinic acetylcholine receptors (nAChRs), disrupting normal cholinergic signaling pathways vital for cognitive functions (26). Chronic exposure induces oxidative damage, leading to cellular damage and neuronal death (26, 33). Additionally, imidacloprid induces neuroinflammation involving glial cell activation, compromising the blood-brain barrier and contributing to neurological dysfunction (33, 34). These mechanisms often act in concert, with nAChR activation leading to oxidative stress and neuroinflammation, ultimately resulting in neuronal apoptosis and impaired plasticity (26, 35).

### 2.2 Metabolic and endocrine disorders

Beyond their recognized neurotoxic effects, neonicotinoid insecticides are increasingly implicated in metabolic and endocrine disruptions, raising significant public health concerns. Research suggests a potential link between exposure to these ubiquitous pesticides and conditions such as obesity, insulin resistance, and thyroid dysfunction, pointing to their role as endocrine-disrupting chemicals (EDC). A growing body of evidence connects neonicotinoid exposure to metabolic disturbances. Experimental studies have shown that neonicotinoids, including imidacloprid, can promote lipid accumulation and contribute to weight gain in animal and *in vitro* models, though extrapolating these findings to human metabolic outcomes requires caution (36, 37). In cross-sectional human studies conducted in South China, neonicotinoids and their metabolites were frequently detected in blood samples, with positive associations observed between these chemicals and high fasting blood glucose and obesity in both normal-weight and obese populations (24). As these are cross-sectional data, they indicate a statistical association but cannot establish temporality or rule out residual confounding; longitudinal studies are needed to assess the direction and independence of this relationship. Experimental models further support these findings, demonstrating that imidacloprid can potentiate high-fat diet-induced adiposity and insulin resistance in male mice, affecting genes regulating lipid and glucose metabolism (38).

Neonicotinoids also appear to interfere with thyroid hormone regulation. *In vitro* evidence indicates that certain neonicotinoids interact with thyroid hormone receptors (39). Animal studies have provided *in vivo* support; for example, thiamethoxam exposure has been shown to increase T3 and T4 levels in lizards, while a mixture of thiacloprid and deltamethrin increased T3 and T4 levels in rats' serum. In addition, animals exposed to imidacloprid reported decreased plasma levels of thyroid hormones (T4 and TSH) (40). The observed metabolic and thyroid-related disturbances align with the classification of neonicotinoids as potential endocrine-disrupting chemicals. Several neonicotinoids, including imidacloprid, clothianidin, and thiamethoxam, have exhibited EDC-like behavior in various studies (41). Laboratory investigations have specifically revealed that imidacloprid can act as an endocrine disruptor by interfering with metabolic homeostasis and steroidogenesis (37). The widespread use and environmental persistence of neonicotinoids underscores their potential threat to public health through metabolic and endocrine disruption. Endocrinologically, thyroid lesions with alterations in luteinizing hormone, follicle-stimulating hormone, and progesterone (42) have been documented in association with exposure to imidacloprid.

### 2.3 Reproductive and immunological risks

Research primarily in animal models indicates that neonicotinoids, including imidacloprid, thiacloprid, and acetamiprid, can adversely affect the reproductive system. These effects manifest as impaired testicular development, damage to spermatogenesis, reduced sperm quality, and altered ovarian morphology (43). For instance, exposure to thiacloprid during gestation has been linked to significant spermatogenic defects in offspring, including increased testis-to-body weight ratios and meiotic errors (44). More broadly, these insecticides often act as endocrine disruptors, interfering with the crucial synthesis, secretion, transport, and action of hormones essential for reproduction and development (45). Neonicotinoids have been shown to increase aromatase expression, an enzyme implicated in breast cancer and developmental processes (46).

Immunological systems are also vulnerable. Studies in mammals demonstrate that imidacloprid can induce immunotoxic effects, altering immune cell populations and function. In male albino rats, oral administration of imidacloprid led to an increase in total leukocyte counts and immunoglobulins but simultaneously decreased phagocytic activity and cellular movement. Histological examinations revealed lymphocytic depletion in the spleen and thymus (47). Similarly, in female BALB/c mice, imidacloprid exposure caused immunotoxic effects over a 28-day period (48). The underlying mechanism involves the interaction of neonicotinoids with nAChRs present on immune cells, which play a role in regulating T cell differentiation, antibody production, and inflammatory responses (49). Beyond direct cellular effects, thiacloprid has been shown to impair antioxidant defenses and increase markers of oxidative stress in immune organs of rats (50). The immune system is also compromised, with significant increases in IL-6 immunopositivity observed (51).

#### 2.3.1 Studies in animal and human models

The majority of insights into reproductive and immunological hazards come from animal studies utilizing models such as rats, mice, zebrafish, and chickens. However, direct human data remain limited (24). Despite this, human exposure is widespread; approximately half of the U.S. general population aged 3 years and older had detectable neonicotinoid levels (44). Neonicotinoids and their metabolites have been frequently detected in human blood samples, with higher concentrations observed in females (24).

#### 2.3.2 Transgenerational impacts

A particularly alarming aspect is the potential for transgenerational effects, where health consequences extend to subsequent generations without direct exposure through epigenetic changes. Preliminary evidence from a single animal study suggests that thiacloprid may induce transgenerational epigenetic effects through ancestral embryonic paternal exposure, specifically reporting alterations in sperm DNA methylation patterns in subsequent generations (44). These findings should be interpreted with considerable caution, as they are based on a single, unreplicated rodent study, and the field of transgenerational epigenetic inheritance in mammals remains scientifically contested. Central unresolved questions include whether environmentally induced epigenetic marks can reliably survive the two major germline reprogramming events during gametogenesis and early embryogenesis, the extent to which such effects are species-specific, and the degree to which rodent epigenetic inheritance mechanisms are applicable to human biology. Independent replication and mechanistic validation are necessary before these observations can be considered robust evidence of a transgenerational hazard in humans. Fetal and lactational exposure to clothianidin in mice affects the immune system and gut microbiota of both exposed mothers and their offspring (52). Other pesticides have demonstrated minimal effects in directly exposed generations (F0 and F1) but significant adverse outcomes in later generations (F2 and F3), affecting conditions such as prostate disease, obesity, and kidney disease (53).

## 3 Ecohealth: neonicotinoids' far-reaching ecological footprint

The widespread use of neonicotinoid insecticides has raised significant concerns within the ecohealth framework due to their profound and cascading effects on non-target organisms, ecosystem services, and potentially human wellbeing. These systemic pesticides move beyond their application sites, leading to pervasive environmental contamination with implications for biodiversity, food security, and the integrated health of ecosystems.

### 3.1 Effects on pollinators, birds, and biodiversity

Neonicotinoids pose a substantial threat to global biodiversity, particularly impacting terrestrial and aquatic invertebrates, as well as vertebrates like birds (54). Pollinators, such as wild bees and butterflies, are among the most affected, with numerous studies correlating neonicotinoid use with their population declines (55). These insecticides disrupt trophic interactions and reduce food resources, consequently affecting a wide array of species (54). Birds, especially insectivorous species, experience significant negative indirect effects from neonicotinoid use, primarily through the reduction of their insect food supply (56). Research has demonstrated that bird declines in agroecosystems are linked to neonicotinoid application (54). Beyond insects, neonicotinoids also detrimentally affect predators and parasitoids of pests, undermining integrated pest control efforts (55).

On the other hand, the study of neonicotinoids and their impact on aquatic fauna is an emerging topic, given the presence of these insecticides in bodies of water such as rivers and seas. This is due to the use of neonicotinoids in agriculture, which are carried to water bodies by runoff. For example, an 83% decrease in average zooplankton biomass was reported in spring, causing the smelt harvest to drop from 240 to 22 tons in Lake Shinji, Japan (57).

### 3.2 Bioaccumulation and implications for food security

The systemic nature of neonicotinoids facilitates their spread throughout ecosystems, impacting biodiversity through their propagation within food webs (54). While biomagnification in aquatic ecosystems may be less likely, neonicotinoids can accumulate in the tissues of various organisms and be transferred through terrestrial food webs, impacting higher trophic levels (58, 59).

The ecological disruptions caused by neonicotinoids have direct and critical implications for food security and ecosystem services. Many crops rely on insect pollination, and the widespread use of these insecticides directly threatens these vital pollinators, thereby endangering food security and sustainable agriculture development (12). The pervasive and chronic impacts on global biodiversity are having major negative effects on crucial ecosystem services, such as pollination, which are fundamental to food security and sustainable development (60).

### 3.3 The one health approach as an integrative framework

Addressing the multifaceted challenges posed by neonicotinoids necessitates an integrated, interdisciplinary perspective, such as the “One Health” concept (61). This approach recognizes the interconnectedness of human, animal, and environmental health. Neonicotinoids demonstrate how their cascading effects impact fish and bird populations, threaten agricultural productivity through reduced pollination and soil fertility, and lead to widespread contamination of food sources (55).

## 4 Regulation and risk assessment: a critical look at neonicotinoid oversight

The assessment and regulation of neonicotinoid insecticides present a complex challenge, characterized by the limitations of traditional toxicological testing, the underestimation of chronic and low-dose effects, the pervasive issue of mixtures and cumulative exposure, and significant disparities in regulatory approaches across different global regions. These factors collectively highlight the urgent need for a more comprehensive and precautionary framework to safeguard public and environmental health.

### 4.1 Global Regulatory Landscape

Neonicotinoids such as imidacloprid, clothianidin, thiamethoxam, acetamiprid, and dinotefuran stimulate insect nicotinic receptors, leading to sustained neuronal activation, paralysis, and death (62). International regulatory approaches to neonicotinoids differ significantly across jurisdictions, ranging from precautionary bans to reactive and permissive frameworks, reflecting divergent assessments of the available evidence on their risks (10, 18). In Asia, regulation remains inadequate, with continued widespread use (19, 63).

### 4.2 Limitations of traditional toxicological testing

Traditional toxicological testing protocols, often designed around acute toxicity, have proven inadequate for fully capturing the subtle, long-term impacts of neonicotinoids. These conventional methods typically focus on lethal doses or no observed effect concentrations over short exposure periods, such as 48 or 96 hours (7, 64). This approach inherently underestimates the actual risks posed by neonicotinoids, which can exhibit unexpected and potent effects at very low doses and over extended durations (65).

The “one-substance assumption,” where chemicals are evaluated in isolation, fails to account for the combined effects of multiple substances, a common scenario in real-world environmental exposure (20). Furthermore, early chronic toxicity studies often used non-field-realistic doses or test species that were not representative of the broader spectrum of sensitive organisms or life stages, leading to an underestimation of risk (41). The methodologies, largely unchanged despite the novel characteristics of systemic pesticides, have resulted in flawed conclusions regarding their ecological safety (64).

### 4.3 Underestimation of chronic effects and low doses

A significant failing of past risk assessments is the systematic underestimation of chronic effects and the potency of low-dose neonicotinoid exposure. Research shows that neonicotinoids can have time- dependent and time-cumulative effects, meaning that even minute levels of residues can lead to significant impacts if exposure is sufficiently prolonged (66). For example, studies in sensitive invertebrate species have found that the lethal toxicity of imidacloprid under chronic continuous exposure can be up to 100,000 times greater than under acute exposure conditions, reflecting its time-cumulative mode of action at nicotinic acetylcholine receptors (6, 65). Although direct lethality comparisons do not translate straightforwardly to human risk assessment, this figure is toxicologically significant when considered alongside human biomonitoring data. Imidacloprid and its metabolites have been detected in the urine of general population samples at concentrations ranging from 0.1 to 1.0 μg/L, with higher levels reported in agricultural workers and children (13, 17). Although current regulatory acceptable daily intakes for imidacloprid are set at 0.06–0.08 μg/kg body weight/day in major jurisdictions, the time-cumulative toxicity model suggests that safety thresholds derived from short-term acute studies may not adequately protect against the neurological and metabolic effects associated with chronic low-dose exposure at concentrations already detected in human populations.

This phenomenon is often described as “delayed mortality,” where exposed organisms may not die immediately but experience population declines or disappear completely after weeks of continuous low-level exposure (7). The non-linear dose-response relationships and gene reprogramming effects of neonicotinoids further complicate traditional risk assessment, as they can elicit significant impacts at concentrations previously considered safe (65).

### 4.4 Mixtures and cumulative exposure

In real-world environments, non-target species rarely face a single pesticide in isolation but encounter complex mixtures of multiple neonicotinoids and other pesticides, along with various environmental stressors (8). All neonicotinoids bind to the same nAChRs, implying a cumulative toxic effect, yet risk assessments are typically conducted for each chemical separately (64). This “cocktail effect” can result in additive or potentially synergistic interactions, where the combined toxicity of multiple pesticides equals or exceeds the sum of their individual effects. Studies have documented additive and, in some cases, potentially synergistic effects in mixtures containing neonicotinoids at environmentally relevant concentrations, although the distinction between strict synergism and additivity depends on the specific compounds, concentration ratios, and test organisms involved, and requires further characterization across a broader range of field-realistic scenarios (20, 66).

### 4.5 Restrictive European policies and regulatory gaps

Regulatory approaches vary significantly across the globe. The European Union has adopted a more precautionary stance, leading to more stringent regulations, including bans on the outdoor use of several key neonicotinoids like imidacloprid, clothianidin, and thiamethoxam (18, 19). In contrast, regions such as parts of Latin America demonstrate significant regulatory gaps and more reactive approaches. In Brazil, recent legislative changes have facilitated the commercialization of pesticides that are restricted in other jurisdictions, and these changes have been associated with reports of increased bee mortality (67). Although evidence for a direct causal link between the legislative changes and increased human or ecological exposure outcomes remains limited, this regulatory divergence highlights the broader challenge of achieving coherent global pesticide governance in the absence of internationally binding standards (18, 67). Not all regulatory trajectories are moving toward greater permissiveness: the United States Environmental Protection Agency is conducting registration reviews of several neonicotinoids, including imidacloprid, clothianidin, thiamethoxam, and acetamiprid, under its ongoing Registration Review Program (2023–2025), which includes reassessment of pollinator and human health risk endpoints. The outcomes of these reviews, expected to result in updated risk assessments and potential use restrictions, represent a significant development in the regulatory landscape that will affect one of the largest agricultural markets globally (68).

## 5 Toward safer agriculture: directions for transition

The evidence reviewed in this article highlights an urgent need to reduce reliance on neonicotinoid insecticides and accelerate the transition to pest management approaches that are less harmful to human health and ecosystems. Several alternatives are available, including agroecological practices such as diversified crop rotations and habitat management, biological control agents and biopesticides from microbial sources, pheromone-based monitoring and mating disruption systems, and integrated pest management frameworks that use chemical inputs only as a last resort (20, 69, 70). However, the shift to these approaches faces well-documented barriers, including a absence of field-realistic efficacy data across diverse agroecosystems, the economic costs of system redesign, limited access to training and technical support –especially in low- and middle-income countries –and insufficient regulatory and financial incentives to overcome the path dependency created by decades of systemic insecticide use (71–73). Achieving this transition will require coordinated action across regulatory frameworks, agricultural research investment, farmer support systems, and supply chain governance, with explicit attention to equity and context-specificity in designing and implementing alternative pest management strategies.

## 6 Conclusion

This article set out to examine whether the agronomic utility of neonicotinoids justifies their continued widespread use in light of the accumulated evidence on their risks to human health and ecosystems. Based on the evidence critically appraised in the preceding sections, the authors' position is that it does not. This assessment does not claim that harm to humans is proven beyond doubt, but recognizes that the current evidence base, taken as a whole, is sufficient to justify a precautionary regulatory response that has not yet been implemented in most jurisdictions.

The analysis in this article identifies five specific findings that support this position and extend beyond the general sentiments already established in the literature. First, the principal mammalian metabolite of imidacloprid, desnitro-imidacloprid, exhibits substantially greater potency at mammalian nicotinic acetylcholine receptors than the parent compound, yet regulatory safety thresholds are set only for the parent compound. This omission of metabolite-inclusive risk assessment represents a structural deficiency in current pre-market evaluation frameworks. Second, cross-sectional and experimental evidence consistently links neonicotinoid exposure to neurological, metabolic, endocrine, reproductive, and immunological effects across multiple biological systems, though the evidentiary strength varies among these domains. Neurological associations in humans are the most developed, supported by both mechanistic data and epidemiological studies, though these are predominantly cross-sectional. In contrast, human epidemiological evidence for reproductive, immunological, and transgenerational effects remains limited, with most findings derived from animal models whose direct applicability to humans is constrained by pharmacokinetic and species-specific differences discussed earlier in this manuscript. The policy recommendations in this article should therefore be understood as reflecting a graduated evidentiary hierarchy. They are most firmly grounded in the neurological and metabolic evidence, where converging mechanistic and epidemiological data provide the strongest, though still mainly associative, case for regulatory action. They are precautionary regarding reproductive and immunological effects, where animal model evidence is consistent but direct human data are sparse. They are most tentative regarding transgenerational epigenetic effects, which currently rest on a single unreplicated rodent study in a scientifically contested field, as noted in Section 2.3.2; inclusion of this endpoint in the regulatory agenda reflects the potential severity and irreversibility of multigenerational harm rather than the current robustness of the evidence, and is explicitly contingent on independent replication and mechanistic validation in future research. Third, traditional toxicological testing frameworks systematically underestimate risk by evaluating acute rather than chronic endpoints, individual compounds rather than mixtures, and adult organisms rather than developmental life stages. The time-cumulative toxicity model shows that chronic lethality in sensitive organisms can exceed acute lethality by orders of magnitude at concentrations already detected in environmental monitoring. Fourth, the regulatory divergence between jurisdictions that have restricted key neonicotinoids and those that have not –including the absence of metabolite- specific safety thresholds, inadequate characterization of mixture effects, and the pending rather than concluded status of major regulatory reviews such as the US EPA Registration Review Program –reflects the absence of an internationally coherent, evidence-based governance framework. Fifth, the ecological disruptions documented in this article, including pollinator decline, aquatic food web collapse, and bird population losses, are not merely biodiversity concerns but upstream determinants of human nutritional security and rural health that are inadequately addressed by risk assessments focused solely on direct human exposure pathways.

These findings collectively indicate that the question posed in this article's Introduction cannot be resolved by the available evidence alone: the data are sufficient to justify precaution but insufficient to establish causation across most endpoints. This evidentiary gap is itself a regulatory finding. In the absence of longitudinal epidemiological studies, metabolite-inclusive risk assessments, mixture toxicology data, and comprehensive human biomonitoring programs required to resolve current uncertainties, the precautionary principle provides the appropriate decision-making framework. The authors recommend that regulatory agencies revise pre-market assessment requirements to include major human metabolites, cumulative mixture effects, and chronic low-dose endpoints; that biomonitoring programs be established to track neonicotinoid and metabolite concentrations in vulnerable populations; and that international regulatory coordination be strengthened to prevent the continued use of compounds in jurisdictions with weaker governance frameworks than those that have restricted them on precautionary grounds.
